# Forward Flow in Patients With Heart Failure and Functional Mitral Regurgitation: The COAPT Trial

**DOI:** 10.1016/j.jscai.2025.102609

**Published:** 2025-03-26

**Authors:** Zachary M. Gertz, Philippe Pibarot, Zhipeng Zhou, Michael J. Schonning, Björn Redfors, Yanru Li, Saibal Kar, D. Scott Lim, Neil J. Weissman, David J. Cohen, JoAnn Lindenfeld, William T. Abraham, Michael J. Mack, Federico M. Asch, Gregg W. Stone

**Affiliations:** a Division of Cardiology, Pauley Heart Center, Virginia Commonwealth University, Richmond, Virginia; b Department of Medicine, Québec Heart and Lung Institute, Laval University, Québec City, Québec, Canada; c Clinical Trials Center, Cardiovascular Research Foundation, New York, New York; d Department of Cardiology, Sahlgrenska University Hospital, Gothenburg, Sweden; e Department of Population Health Sciences, Weill Cornell Medicine, New York, New York; f Department of Molecular and Clinical Medicine, Gothenburg University, Gothenburg, Sweden; g Cardiovascular Institute, Los Robles Regional Medical Center, Thousand Oaks, California; h Bakersfield Heart Hospital, Bakersfield, California; i Division of Cardiology, University of Virginia, Charlottesville, Virginia; j Cardiovascular Core Laboratories, MedStar Health Research Institute, Washington, DC; k St. Francis Hospital & Heart Center, Roslyn, New York; l Advanced Heart Failure, Vanderbilt Heart and Vascular Institute, Nashville, Tennessee; m Department of Medicine, The Ohio State University, Columbus, Ohio; n Department of Physiology and Cell Biology, The Ohio State University, Columbus, Ohio; o Division of Cardiovascular Medicine, The Ohio State University, Columbus, Ohio; p Davis Heart and Lung Research Institute, The Ohio State University, Columbus, Ohio; q Baylor Scott & White Heart and Vascular Hospital, Plano, Texas; r The Zena and Michael A. Wiener Cardiovascular Institute, Icahn School of Medicine at Mount Sinai, New York, New York

**Keywords:** COAPT, echocardiography, functional mitral regurgitation, heart failure, transcatheter edge-to-edge repair

## Abstract

**Background:**

Heart failure (HF) is characterized by a reduction in forward cardiac output (forward flow), potentially worsened by functional mitral regurgitation (FMR). The impact of reduced forward flow in HF patients with FMR is uncertain, and the outcomes of mitral transcatheter edge-to-edge repair (TEER) according to forward flow levels have not been described.

**Methods:**

This study assessed the change in baseline flow in patients with HF and FMR enrolled in the COAPT trial randomized to TEER plus guideline-directed medical therapy (GDMT) compared with GDMT alone. Patients were stratified into tertiles of baseline forward flow using the Doppler-derived stroke volume index. The primary outcome was the composite rate of death or HF hospitalization at 24 months. Clinical, echocardiographic, and outcome measures were assessed.

**Results:**

Among patients randomized to GDMT alone, the lowest baseline forward flow tertile was associated with worse outcomes (*P* = .04). In contrast, baseline forward flow tertile was not associated with outcomes among patients randomized to TEER + GDMT (*P* = .88). Patients in the lowest tertile treated with TEER + GDMT had the largest absolute reduction in the primary outcome (44.6% vs 75.7%; hazard ratio [HR], 0.43; 95% CI, 0.29-0.63), whereas patients in the highest tertile had the smallest absolute benefit after TEER (42.8% vs 57.9%; HR, 0.69; 95% CI, 0.45-1.04). However, the relative treatment effect was not different between tertiles (p_interaction_ = 0.32). Mean forward flow did not significantly increase during 2-year follow-up, and was similar between treatment groups at all time periods.

**Conclusions:**

In the COAPT trial, lower baseline forward flow was associated with worse outcomes in medically managed patients, and those with low baseline forward flow derived the greatest absolute benefit from TEER. However, measured forward flow did not improve with TEER during the 2-year follow-up.

## Introduction

In patients with heart failure (HF) and reduced left ventricular ejection fraction (LVEF), decreased contractility can result in reduced forward stroke volume and cardiac output. Similarly, retrograde blood flow into the left atrium due to mitral regurgitation (MR) may also cause a reduction in forward blood flow. Functional MR (FMR) in HF occurs as a result of left ventricular (LV) remodeling, with displacement of the papillary muscles and a reduction in closing forces.^1^ In patients with reduced LVEF, the extent to which FMR further reduces forward flow and whether forward flow improves after MR is treated is not known. A better understanding of forward flow in patients with FMR, before and after treatment with transcatheter edge-to-edge repair (TEER), could lead to improved patient assessment and risk stratification, inform management, and possibly assist in evaluating treatment success.

The COAPT (Cardiovascular Outcomes Assessment of the MitraClip Percutaneous Therapy for Heart Failure Patients with Functional Mitral Regurgitation) trial (ClinicalTrials.gov identifier: NCT01626079) demonstrated that TEER with the MitraClip device (Abbott) reduces HF hospitalization (HFH) and mortality in selected HF patients with severe FMR.^2^ We performed a subanalysis from the COAPT trial, designed to assess the following: (1) the association between baseline forward flow and outcomes in patients in the COAPT trial; (2) the magnitude of changes in forward flow after TEER; and (3) whether those changes are associated with patient outcomes.

## Methods

### Study design

Details of the COAPT trial protocol and design have been previously published.^2^ The COAPT trial was approved by the institutional review board at each site, and written informed consent was obtained from all patients. Briefly, COAPT was a multicenter, randomized, controlled, open-label clinical trial in patients with HF and moderate-to-severe (3+) or severe (4+) FMR who remained symptomatic despite maximally tolerated guideline-directed medical therapy (GDMT). Inclusion criteria required LVEF 20% to 50% and LV end-systolic dimension ≤70 mm. Exclusion criteria included severe pulmonary hypertension, severe tricuspid regurgitation requiring surgery or intervention, and moderate or severe right ventricular dysfunction, among others. Heart teams at the participating centers identified patients with mitral-valve anatomy suitable for TEER, and eligibility for inclusion in the trial was confirmed by a Central Eligibility Committee. Approved patients were randomized in a 1:1 ratio to either TEER with the MitraClip device plus GDMT or GDMT alone. Follow-up in COAPT was performed through 5 years, but for the present analysis, data were analyzed through 24 months as persistently symptomatic control group patients were allowed to crossover to MitraClip device after this time. In addition, only those patients for whom baseline forward stroke volume index (SVI) could be calculated were included in the present analysis.

### Echocardiographic core laboratory analysis

Transthoracic echocardiograms were performed at baseline and at 1, 6, 12, 18, and 24 months after randomization. Echocardiographic findings were assessed by an independent echocardiographic core laboratory (MedStar Health Research Institute) as previously described.^3^ The primary measure of forward flow was forward SVI. Forward SVI was calculated by Doppler using the velocity-time integral of the LV outflow tract and LV diameter in midsystole in the parasternal long-axis view, divided by body surface area. Forward SVI was calculated for all echocardiograms through 24 months. Other echo parameters were measured as previously described.^3^

### Study end points

The principal end point of interest was the time to the first occurrence of all-cause mortality or HFH within 2 years. Additional analyses were performed to assess secondary end points that have been previously described.^2^ Adverse events were adjudicated by an independent clinical events committee. We also evaluated the association of the change in forward SVI from baseline to 30 days stratified by treatment group with subsequent clinical outcomes from 30 days to 2 years.

### Statistical analyses

Normally distributed continuous data are expressed as mean ± SD and were compared with *t* tests. Nonnormally distributed continuous data are expressed as median (IQR) and were compared using the Wilcoxon rank sum test. Categorical variables are summarized as percentages and were compared using the χ^2^ test or Fisher exact test as appropriate. Time-to-event variables are summarized as Kaplan-Meier event rates and were compared across the SVI tertiles by the log-rank test. Hazard ratios and 95% CIs were determined using Cox proportional hazards regression. Interaction testing was done to assess whether the relative hazards between baseline forward flow and outcomes varied by treatment group. We also used spline regression with knots at 22.3 mL/m^2^ and 30.1 mL/m^2^ to assess whether there was a nonlinear relationship between baseline forward SVI and clinical end points. Changes in echocardiographic parameters over time were calculated as the difference between the baseline and follow-up visits. Analysis of covariance was performed to compare changes over time adjusted for baseline values. Subjects without an available follow-up echocardiographic image who had an adjudicated HF-related death prior to that visit were assigned the worst change from baseline to that visit. For all other subjects who had missing echocardiographic values due to other reasons (eg, death not due to HF, withdrawals, missing echoes, and so on), multiple imputations with Markov Chain Monte Carlo were used with a monotone missing pattern. A 2-sided *P* value <.05 was considered statistically significant for all superiority tests. All statistical analyses were performed with SAS software, version 9.4 (SAS Institute). The data that support the findings of this report may be made available to qualified investigators upon reasonable request. Such requests should be made to Gregg W. Stone (gregg.stone@mountsinai.org).

## Results

### Patient characteristics

Of the 614 patients included in the COAPT trial, baseline forward SVI was analyzable in the echocardiographic core laboratory in 568 (92.5%). Patients were stratified by baseline forward SVI into tertiles, with medians (IQR) of 18.4 (15.9-20.2) mL/m^2^ (first tertile, n = 189), 25.6 (24.0-28.0) mL/m^2^ (second tertile, n = 190) and 34.9 (32.3-39.2) mL/m^2^ (third tertile, n = 189). The proportion of patients randomized to TEER was similar in all tertiles (n = 100 [52.9%] vs n = 95 [50.0%] vs n = 87 [46.0%]; *P* = .41) (Supplemental Figure S1). The baseline clinical characteristics of the study groups are shown in Supplemental Table S1. Patients with lower baseline forward SVI were younger and less often White. They were less likely to have ischemic HF etiology. Female sex, body surface area, and presence of atrial fibrillation were similar between groups.

### Echocardiographic characteristics

Baseline echocardiographic characteristics stratified by tertile of forward SVI are shown in Supplemental Table S2. Patients with lower baseline forward SVI had larger left ventricles with lower LVEF. Additionally, patients with lower forward SVI were more likely to have severe (4+) MR.

### Patient outcomes

**Figure 1:**
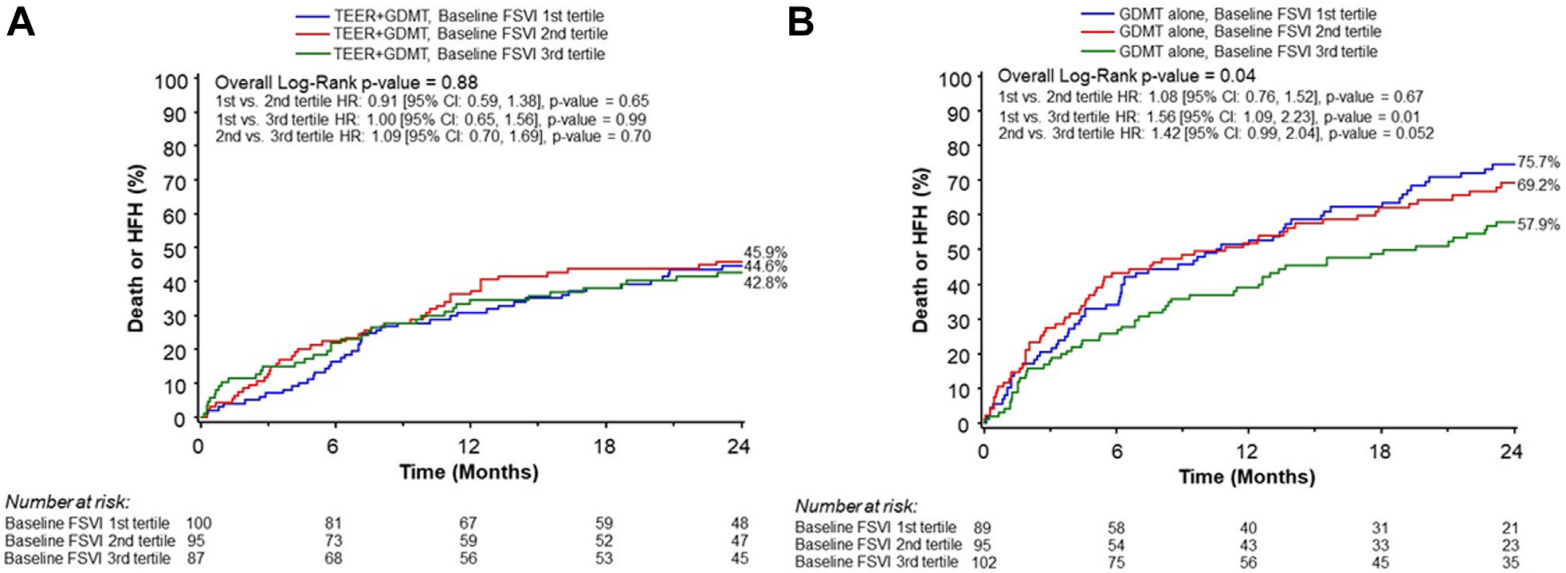
**Primary outcome stratified by forward flow and treatment group.** Kaplan-Meier curves showing the primary composite outcome of death or heart failure hospitalization (HFH) during 2-year follow-up, in patients randomized to transcatheter edge-to-edge repair (TEER) plus guideline-directed medical therapy (GDMT) (A) or GDMT alone (B), stratified by tertile of baseline forward stroke volume index (FSVI). HR, hazard ratio.

**Figure 2:**
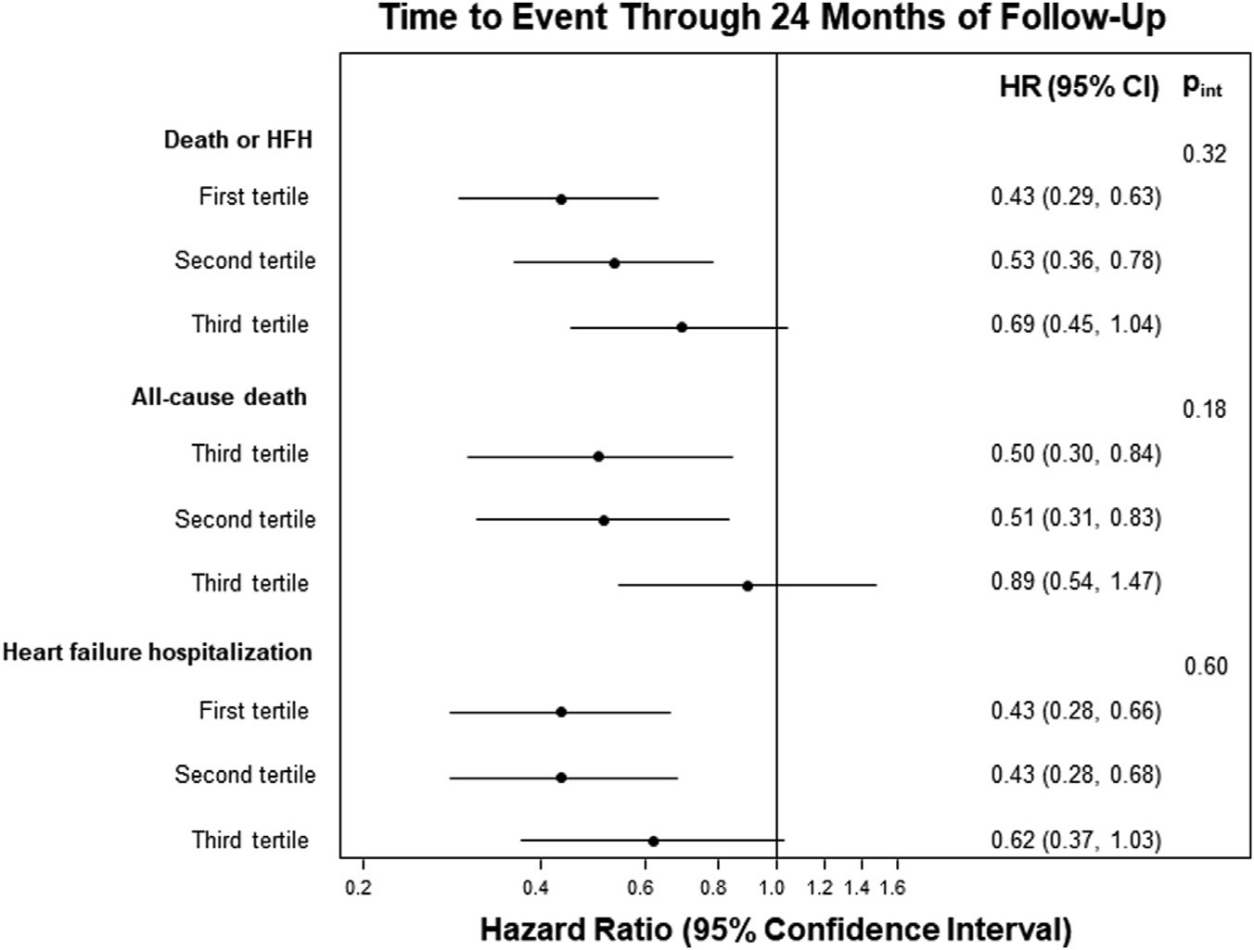
**Hazard ratios (HR) for death, heart failure hospitalization (HFH), and death or HFH at 24 months.** Impact of transcatheter edge-to-edge repair on the primary outcome and its components stratified by baseline forward stroke volume index tertile.

**Figure 3:**
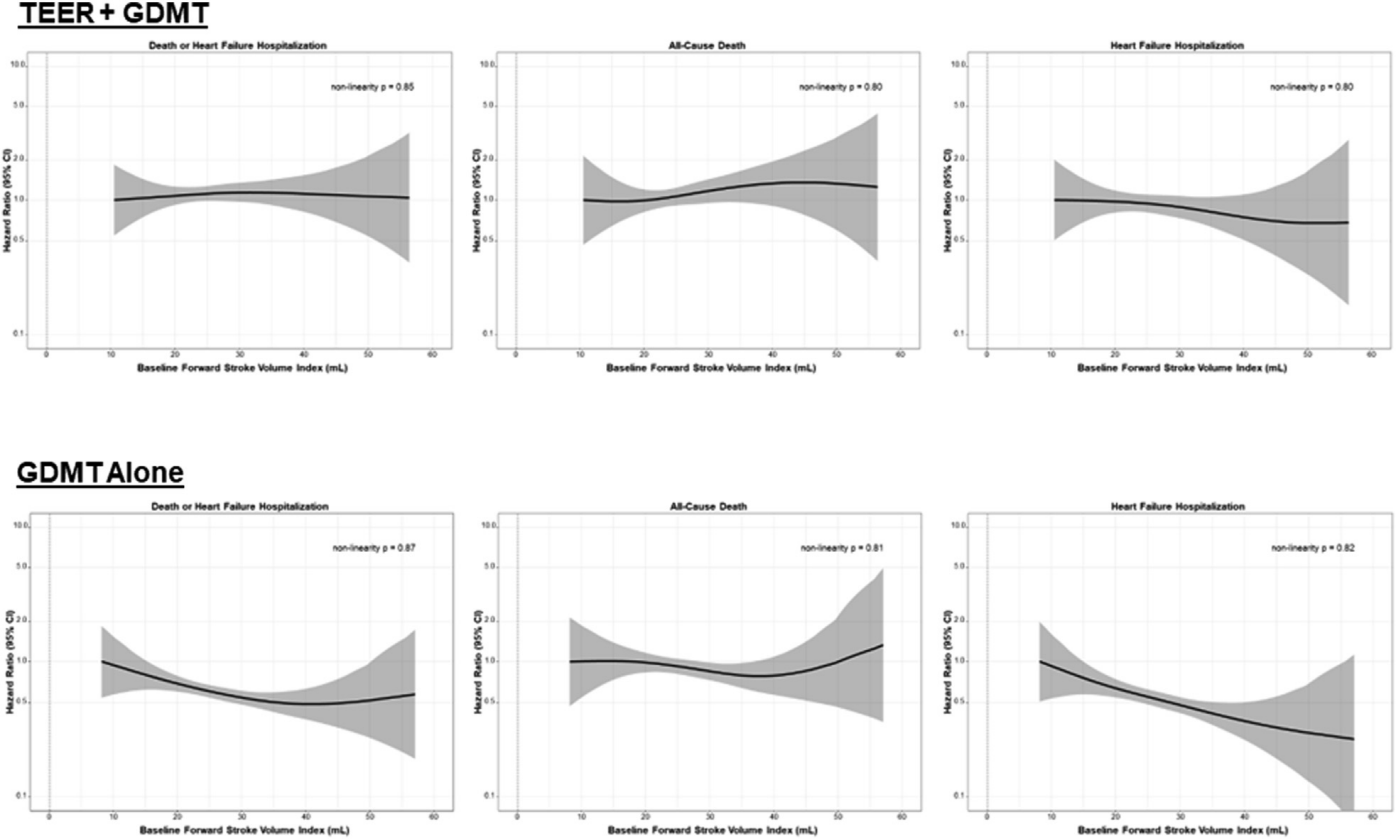
**Spline curves for clinical outcomes in the transcatheter edge-to-edge repair (TEER) plus guideline-directed medical therapy (GDMT)(top row) and GDMT alone (bottom row) groups.**

The tertile of baseline forward SVI was significantly associated with the primary outcome of death or HFH at 2 years among patients randomized to medical therapy (*P* = .04), but not among those randomized to TEER (*P* = .88) (p_interaction_ = 0.32) (Figure 1, Central Illustration). Among patients randomized to medical therapy, those with lower baseline forward flow had higher rates of the primary outcome compared with those with higher forward flow. The primary and secondary outcomes, stratified by forward SVI tertile and treatment group, are shown in Table 1. The relative benefits of TEER were consistent across all SVI tertiles. The absolute improvements in the 2-year rates of death or HFH with TEER treatment compared with GDMT alone increased from the lowest to the mid to the highest baseline SVI tertile (lowest tertile: 44.6% vs 75.7%, *P* < .001; middle tertile: 45.9% vs 69.2%, *P* = .001; highest tertile: 42.8 % vs 57.9%, *P* = .08) (Figure 2). However, the *P* value for interaction between baseline forward flow and the relative benefit of TEER treatment for the 2-year outcome of death or HFH was 0.32. By spline analysis, the associations between baseline forward SVI and clinical outcomes were linear, nonsignificant, and independent of treatment assignment (Figure 3).

**Central Illustration:**
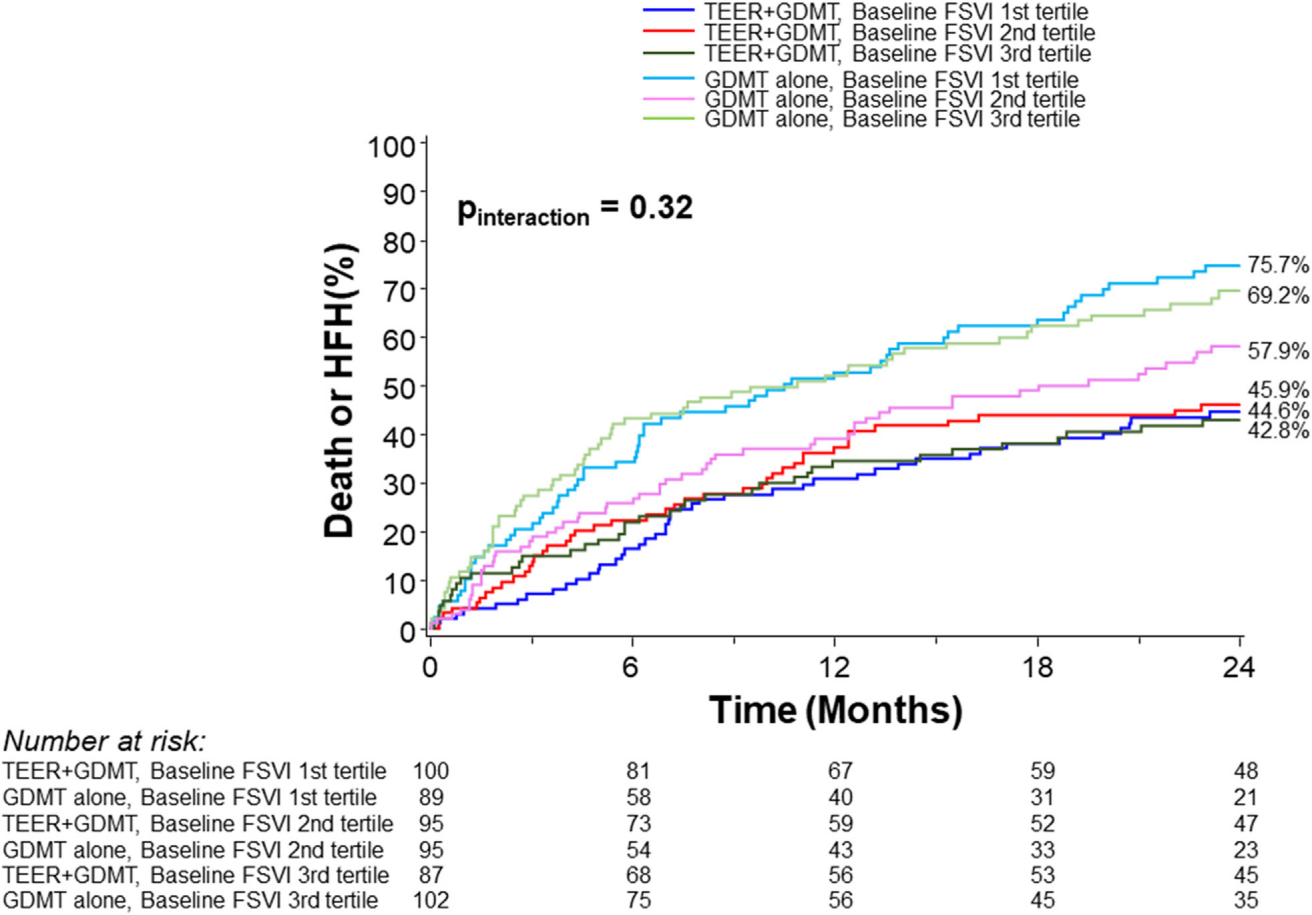
Kaplan-Meier curves showing the primary composite outcome of death or heart failure hospitalization (HFH) in patients randomized to transcatheter edge-to-edge repair (TEER) plus guideline-directed medical therapy (GDMT) or GDMT alone, stratified by tertile of baseline forward stroke volume index (FSVI).

**Table 1:** Primary and secondary outcomes according to baseline forward flow tertile and treatment group.

	First tertile n = 189			Second tertile n = 190			Third tertile n = 189			*P* value for interaction
	TEER + GDMT	GDMT Alone	HR (95% CI)	TEER + GDMT	GDMT Alone	HR (95% CI)	TEER + GDMT	GDMT Alone	HR (95% CI)	
Death or HFH	43 (44.6)	65 (75.7)	0.43 (0.29-0.63)	43 (45.9)	64 (69.2)	0.53 (0.36-0.78)	37 (42.8)	56 (57.9)	0.69 (0.45-1.04)	.32
Death from CV cause or HFH	43 (44.6)	63 (4.6)	0.44 (0.30-0.65)	38 (42.4)	58 (64.0)	0.52 (0.35-0.79)	33 (39.1)	50 (52.8)	0.69 (0.45-1.07)	.40
All-cause death	23 (24.1)	36 (43.3)	0.50 (0.30-0.84)	25 (26.8)	43 (48.9)	0.51 (0.31-0.83)	28 (32.5)	35 (37.3)	0.89 (0.54-1.47)	.18
CV cause	21 (22.1)	32 (39.3)	0.51 (0.30-0.89)	19 (21.5)	32 (39.2)	0.52 (0.29-0.91)	22 (26.8)	27 (29.8)	0.91 (0.52-1.60)	.27
Related to HF	12 (13.5)	20 (26.5)	0.47 (0.23-0.96)	7 (8.8)	25 (32.6)	0.24 (0.10-0.55)	9 (11.8)	13 (15.1)	0.78 (0.33-1.81)	.17
Not related to HF	9 (9.9)	12 (17.4)	0.59 (0.25-1.40)	12 (13.9)	7 (9.8)	1.54 (0.61-3.92)	13 (17.0)	14 (17.3)	1.03 (0.48-2.18)	.33
Non-CV cause	2 (2.6)	4 (6.5)	0.38 (0.07-2.08)	6 (6.8)	11 (16.0)	0.49 (0.18-1.32)	6 (7.8)	8 (10.7)	0.84 (0.29-2.43)	.65
All-cause hospitalization	61 (62.9)	73 (88.1)	0.52 (0.37-0.74)	63 (69.5)	70 (78.3)	0.79 (0.56-1.10)	60 (71.4)	77 (79.3)	0.89 (0.64-1.25)	.10
CV cause	51 (53.0)	60 (74.9)	0.60 (0.41-0.87)	43 (51.5)	61 (69.2)	0.58 (0.39-0.85)	37 (45.1)	54 (57.7)	0.72 (0.47-1.09)	.77
Related to HF	36 (39.2)	54 (68.5)	0.43 (0.28-0.66)	29 (34.9)	53 (60.9)	0.43 (0.28-0.68)	23 (28.5)	40 (43.0)	0.62 (0.37-1.03)	.60
Not related to HF	25 (26.9)	19 (27.0)	1.13 (0.62-2.05)	24 (30.5)	24 (32.3)	0.89 (0.51-1.57)	21 (28.4)	29 (34.9)	0.79 (0.45-1.38)	.72
Major bleeding	4 (4.0)	2 (2.3)	1.80 (0.33-9.80)	1 (1.1)	1 (1.1)	1.04 (0.06-16.60)	10 (12.2)	0 (0)	N/A	.95
Unplanned mitral-valve intervention	2 (2.0)	3 (5.1)	0.54 (0.09-3.21)	4 (4.8)	5 (10.3)	0.65 (0.17-2.46)	4 (5.8)	8 (11.0)	0.54 (0.16-1.78)	.96
MitraClip implantation	2 (2.0)	1 (2.3)	1.59 (0.14-17.63)	3 (3.6)	4 (9.1)	0.58 (0.13-2.62)	4 (5.8)	5 (8.0)	0.84 (0.22-3.12)	.81
Mitral-valve surgery	0 (0)	2 (2.8)	N/A	1 (1.2)	1 (1.1)	1.01 (0.06-16.16)	0 (0)	3 (3.0)	N/A	1.00
PCI or CABG	1 (1.1)	1 (1.4)	0.82 (0.05-13.06)	1 (1.4)	3 (4.8)	0.28 (0.03-2.71)	4 (5.5)	5 (5.7)	0.91 (0.24-3.40)	.70
PCI	1 (1.1)	1 (1.4)	0.82 (0.05-13.06)	1 (1.4)	3 (4.8)	0.28 (0.03-2.71)	4 (5.5)	4 (4.7)	1.13 (0.28-4.53)	.61
CABG	0 (0)	0 (0)	N/A	0 (0)	0 (0)	N/A	0 (0)	1 (1.0)	N/A	1.00
Neurological event	5 (5.5)	7 (11.0)	0.56 (0.18-1.77)	4 (4.9)	4 (5.9)	0.91 (0.23-3.63)	3 (4.0)	8 (9.4)	0/42 (0.11-1.59)	.73
Stroke	4 (4.5)	5 (8.0)	0.63 (0.17-2.33)	3 (3.9)	4 (5.9)	0.66 (0.15-2.96)	3 (4.0)	6 (7.3)	0.56 (0.14-2.25)	.98
TIA	1 (1.0)	2 (3.2)	0.40 (0.04-4.43)	2 (2.5)	0 (0)	N/A	0 (0)	2 (2.1)	N/A	1.00
Myocardial infarction	3 (3.3)	5 (7.4)	0.48 (0.12-2.03)	3 (3.7)	5 (8.0)	0.51 (0.12-2.16)	7 (9.2)	5 (5.9)	1.62 (0.52-5.12)	.33
New CRT	1 (1.2)	3 (4.7)	0.26 (0.03-2.49)	3 (3.9)	1 (1.1)	2.56 (0.26-24.85)	1 (1.2)	4 (4.2)	0.30 (0.03-2.66)	.27
LVAD implantation or heart transplantation	5 (6.0)	11 (16.1)	0.34 (0.12-0.99)	3 (4.2)	6 (7.6)	0.44 (0.11-1.78)	1 (1.7)	4 (4.2)	0.28 (0.03-2.49)	.93
LVAD implantation	4 (4.9)	8 (11.7)	0.38 (0.12-1.28)	1 (1.4)	4 (5.0)	0.23 (0.03-2.05)	1 (1.7)	3 (3.2)	0.37 (0.04-3.55)	.91
Heart transplantation	1 (1.2)	4 (5.7)	0.20 (0.02-1.76)	2 (2.8)	3 (4.7)	0.56 (0.09-3.36)	0 (0)	1 (1.0)	N/A	.74
Renal replacement therapy	1 (1.2)	8 (11.9)	0.09 (0.01-0.74)	3 (4.1)	6 (8.3)	0.44 (0.11-1.77)	2 (2.7)	5 (7.0)	0.43 (0.08-2.22)	.41

Data are presented as events (Kaplan-Meier %).

CABG, coronary artery bypass grafting; CRT, cardiac resynchronization therapy; CV, cardiovascular; GDMT, guideline-directed medical therapy; HF, heart failure; HFH, heart failure hospitalization; LVAD, left ventricular assist device; PCI, percutaneous coronary intervention; TEER, transcatheter-edge-to-edge repair; TIA, transient ischemic attack.

### Changes in forward flow over time

Follow-up echocardiograms with calculated forward SVI at 30 days were available in 461 patients. From baseline to 30 days, forward SVI increased in 231 (50.1%) patients and decreased or remained unchanged in 230 (49.9%) patients. Clinical and echocardiographic characteristics of those with increased vs unchanged/decreased forward SVI are shown in Supplemental Tables S3 and S4. Patients with increased forward SVI at 30 days were more likely to be male, and to have presented with lower baseline forward flow and more severe FMR at baseline. The median changes in baseline forward flow through 30 days in patients treated with TEER plus GDMT vs GDMT alone were 1.0 (–5.4 to 5.5) mL and –0.2 (–6.1 to 5.2) mL respectively, *P* = .61.

Patients with increased forward SVI from baseline to 30 days were less likely to die (26.7% vs 35.7%, *P* = .04) or undergo heart transplantation (0% vs 3.1%, *P* = .01) between 30 days and 2 years but otherwise had similar outcomes as patients without forward flow improvement (Table 2). There were no significant interactions between improvement in forward flow from baseline to 30 days and treatment group on outcomes between 30 days and 2 years (Supplemental Table S5).

**Table 2:** Adverse events between 30 days and 2 years stratified by whether forward flow increased from baseline to 30 days.

	Increase (n = 231)	No change or decrease (n = 230)	*P* value
Death or hospitalization from HF	115 (50.6)	127 (56.1)	.18
Death from CV cause or HFH	110 (49.0)	115 (52.0)	.38
All-cause death	59 (26.7)	79 (35.7)	.04
CV cause	50 (23.2)	61 (29.0)	.18
Related to HF	29 (14.3)	34 (17.3)	.40
Not related to HF	21 (10.4)	27 (14.1)	.28
Non-CV cause	9 (4.5)	18 (9.5)	.06
All-cause hospitalization	163 (72.3)	168 (75.2)	.10
CV cause	124 (55.8)	124 (57.8)	.40
Related to HF	96 (44.1)	94 (44.2)	.73
Not related to HF	53 (26.1)	64 (32.6)	.13
Death or hospitalization from HF	115 (50.6)	127 (56.1)	.18
Death from CV cause or HFH	110 (49.0)	115 (52.0)	.38
Major bleeding	4 (1.8)	9 (4.1)	.15
Unplanned mitral valve intervention	8 (4.4)	11 (5.6)	.40
MitraClip implantation	6 (3.5)	8 (4.3)	.50
Mitral-valve surgery	2 (1.0)	3 (1.3)	.63
PCI or CABG	3 (1.6)	6 (3.3)	.27
PCI	3 (1.6)	6 (3.3)	.27
CABG	0 (0)	0 (0)	–
Neurological event	13 (6.9)	14 (6.9)	.76
Stroke	9 (4.9)	12 (6.1)	.44
Transient ischemic attack	4 (2.0)	3 (1.4)	.73
Myocardial infarction	10 (5.1)	15 (7.5)	.26
New cardiac resynchronization therapy	8 (4.1)	4 (1.8)	.27
LVAD implantation or heart transplantation	7 (3.9)	14 (7.1)	.09
LVAD implantation	7 (3.9)	9 (4.6)	.54
Heart transplantation	0 (0)	6 (3.1)	.01
Renal replacement therapy	12 (6.5)	9 (4.8)	.56

Data are presented as events (Kaplan-Meier %).

CABG, coronary artery bypass grafting; CV, cardiovascular; HF, heart failure; HFH, heart failure hospitalization; LVAD, left ventricular assist device; PCI, percutaneous coronary intervention.

**Figure 4:**
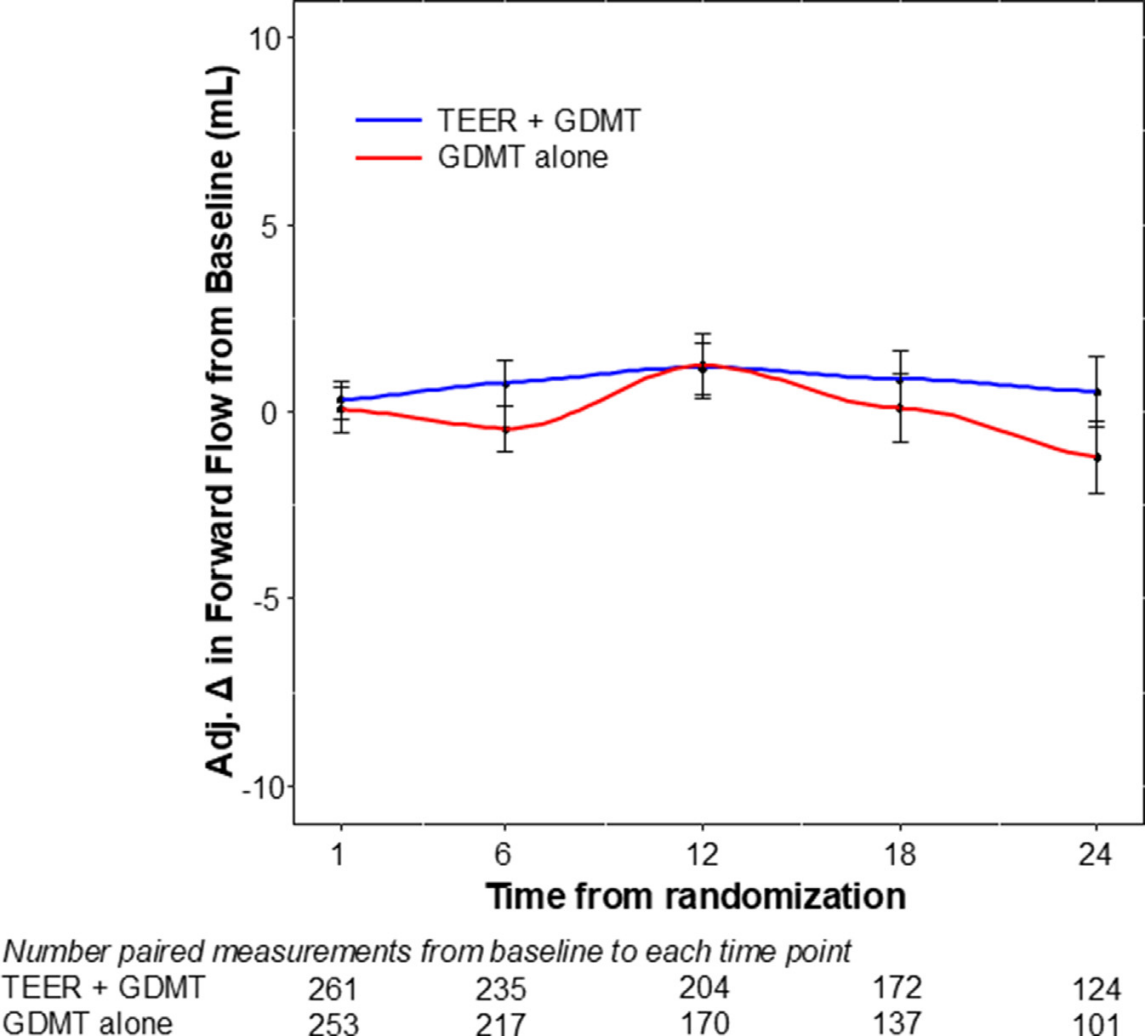
**Imputed forward flow over time stratified by treatment group.** Forward stroke volume index at baseline and at time points through 24 months, for the transcatheter edge-to-edge repair (TEER) plus guideline-directed medical therapy (GDMT) and GDMT alone groups. At no time point did the between-group difference, or the difference compared to baseline, reach statistical significance (*P* > .05 for all comparisons).

During the 2-year follow-up, mean forward SVI was unchanged from baseline in both treatment groups, with no significant differences between groups at any time point (Figure 4). These findings were similar when imputation for missing data was not performed (Supplemental Figure S2).

## Discussion

We conducted a subanalysis from the COAPT trial evaluating the association between baseline forward SVI and outcomes, stratified by treatment group. Our study has several notable findings: (1) patients with low baseline forward SVI had unique clinical and echocardiographic characteristics compared with patients with higher baseline forward SVI; (2) baseline forward flow was significantly associated with outcomes in medically managed patients, but not in those treated with TEER; (3) the relative benefits of TEER were similar across tertiles of baseline forward SVI, although the greatest absolute benefits of TEER were observed in those with the lowest forward SVI rates at baseline; and (4) treatment with TEER did not significantly improve forward SVI at any time during the 2-year follow-up period compared with GDMT alone, but those patients in both groups with improved forward SVI from baseline to 30 days had lower rates of death and heart transplantation between 30 days and 2 years.

Heart failure is characterized by a reduction in cardiac output. Nonetheless, despite its ease of measurement, forward flow has not been analyzed nor emphasized in most large, prospective studies of patients with HF. In a retrospective analysis of hospitalized HF patients with reduced LVEF, reduced SVI was associated with worse outcomes after controlling for other variables, including LVEF.^4^ A large retrospective study of patients treated at Veterans Affairs hospitals showed that SVI derived from right heart catheterization was associated with long-term mortality, also after adjusting for LVEF.^5^ Measures of forward flow have been more thoroughly assessed in studies of valvular heart disease. Low flow is a well-established independent marker of outcomes in patients with aortic stenosis.^6^ Notably, the usual threshold for low flow in aortic stenosis, 35 mL/m^2^, is higher than that seen for nearly all patients enrolled in the COAPT trial.

In patients undergoing surgery for primary MR, forward LVEF and SVI are strongly correlated with outcomes,^7^ while indexed forward LVEF also correlates with postoperative LVEF.^8^ In prior studies of FMR that have included forward flow, this measure has been shown to correlate with other metrics of LV function. In an analysis of patients with dilated nonischemic cardiomyopathy, MR severity was significantly associated with measures of forward flow, and forward LVEF correlated with global longitudinal strain, regardless of MR severity.^9^ In the COAPT trial, forward SVI was strongly correlated with the 2-year composite rate of death or HFH and each of its individual components in medically managed patients. This relationship was not present after TEER, suggesting that treatment of severe FMR may mitigate the adverse impact of reduced forward flow on prognosis. However, the interaction between baseline forward flow and treatment on the relative improvement in the 2-year rates of death or HFH was negative, warranting a cautious interpretation. Still, although all patients benefited from TEER regardless of baseline SVI, the greatest absolute improvements in death or HFH after TEER were noted in the patients with the lowest baseline SVI. Further studies of forward flow and its prognostic impact in HF patients with severe FMR are warranted.

Perhaps surprisingly, we did not find a significant increase in forward SVI in patients treated with TEER compared to GDMT alone at any time during the 2-year follow-up period. It is intuitive to believe that blood that is no longer regurgitated into the left atrium after correction of MR will instead flow forward, but this may not be the case because of increased afterload to LV ejection after MR correction. In the echocardiographic analysis from the COAPT trial, LVEF at 1 month was higher in the control group than after TEER, but at 2 years LVEF in both groups was similar.^3^ Patients with FMR included in the Sentinel registry had no significant changes in LV geometry or LVEF 1 year after TEER.^10^ Patients with FMR treated with the MitraClip device in the EVEREST II trial had reductions in LV dimensions but similar LVEF after 1 year.^11^ Thus, reducing regurgitant blood flow in patients with severe FMR may not automatically translate into higher forward flow or improved LVEF.

In contrast, some uncontrolled case series have suggested that forward SVI improves after TEER.^12^^,^^13^ These findings are not necessarily discordant with our study. In the case series by Kubo et al,^12^ just under half of the patients with FMR were considered responders, with at least a 9% increase in forward SVI. In our study, just over half of patients had an increased forward SVI at 1 month, with an average improvement of 30%. Importantly though, this change was independent of the treatment arm, demonstrating that medically managed patients were just as likely to have improvements in flow as those treated with TEER. In the absence of thorough echocardiographic follow-up from the Mitra-FR trial,^14^ the COAPT trial may provide the best opportunity to examine the impact of TEER on LV performance with comparisons to an appropriate control population.

### Limitations

Several limitations of our study must be noted. Our results cannot be generalized to patients who do not meet the inclusion and exclusion criteria of the COAPT trial. Forward SVI could not be measured in a small subset of the population. By separating into treatment groups and then subdividing into tertiles of forward SVI, our study may have been underpowered to detect differences in some outcomes or between treatments, as suggested by the negative interaction values, despite the apparently varying relationship between baseline forward flow and treatment on outcomes. Finally, to preserve the intention-to-treat randomization, the present analysis was truncated at the 2-year follow-up. However, the nearly identical changes in forward SVI between treatment groups at multiple time points through 2 years suggest that the results would not differ with later assessments.

## Conclusion

In the present substudy from the COAPT trial, Doppler-derived forward flow was associated with 2-year patient outcomes in medically managed patients with HF and severe FMR. TEER improved the prognosis of patients with HF and severe FMR at all degrees of baseline forward flow, although the greatest absolute benefits were noted in those with the worst forward flow at baseline. Despite the markedly improved prognosis of patients treated with TEER in the COAPT trial and the observed association between improved forward flow and subsequent survival, forward flow was not improved by treatment with TEER compared with GDMT alone. Given its ease of measurement and prognostic relevance, future studies of patients with HF and FMR should consider routine assessment of forward SVI (and its incorporation for the measurement of regurgitant fraction) in patients treated medically and with novel HF and FMR therapies.

## References

[1] He S., Fontaine A.A., Schwammenthal E., Yoganathan A.P., Levine R.A. (1997). Integrated mechanism for functional mitral regurgitation: leaflet restriction versus coapting force: in vitro studies. Circulation.

[2] Stone G.W., Lindenfeld J., Abraham W.T. (2018). Transcatheter mitral-valve repair in patients with heart failure. N Engl J Med.

[3] Asch F.M., Grayburn P.A., Siegel R.J. (2019). Echocardiographic outcomes after transcatheter leaflet approximation in patients with secondary mitral regurgitation: the COAPT trial. J Am Coll Cardiol.

[4] Mele D., Pestelli G., Dal Molin D.D. (2020). Echocardiographic evaluation of left ventricular output in patients with heart failure: a per-beat or per-minute approach?. J Am Soc Echocardiogr.

[5] Bavry A.A., Hess E., W Waldo S. (2020). Long-term predictive value of stroke volume index obtained from right heart catheterization: insights from the Veterans Affairs clinical assessment, reporting, and tracking program. Clin Cardiol.

[6] Herrmann H.C., Pibarot P., Hueter I. (2013). Predictors of mortality and outcomes of therapy in low-flow severe aortic stenosis: a Placement of Aortic transcatheter Valves (PARTNER) trial analysis. Circulation.

[7] Dupuis M., Mahjoub H., Clavel M.A. (2017). Forward left ventricular Ejection Fraction: a simple risk marker in patients with primary mitral regurgitation. J Am Heart Assoc.

[8] Magne J., Szymanski C., Fournier A., Malaquin D., Avierinos J.F., Tribouilloy C. (2015). Clinical and prognostic impact of a new left ventricular ejection index in primary mitral regurgitation because of mitral valve prolapse. Circ Cardiovasc Imaging.

[9] Kamperidis V., Marsan N.A., Delgado V., Bax J.J. (2016). Left ventricular systolic function assessment in secondary mitral regurgitation: left ventricular ejection fraction vs. speckle tracking global longitudinal strain. Eur Heart J.

[10] Nickenig G., Estevez-Loureiro R., Franzen O. (2014). Percutaneous mitral valve edge-to-edge repair: in-hospital results and 1-year follow-up of 628 patients of the 2011-2012 Pilot European Sentinel Registry. J Am Coll Cardiol.

[11] Ailawadi G., Lim D.S., Mack M.J. (2019). One-year outcomes after MitraClip for functional mitral regurgitation. Circulation.

[12] Giannini C., Petronio A.S., De Carlo M. (2014). Integrated reverse left and right ventricular remodelling after MitraClip implantation in functional mitral regurgitation: an echocardiographic study. Eur Heart J Cardiovasc Imaging.

[13] Kubo S., Nakamura M., Shiota T. (2017). Impact of forward stroke volume response on clinical and structural outcomes after percutaneous mitral valve repair with MitraClip. Circ Cardiovasc Interv.

[14] Obadia J.F., Messika-Zeitoun D., Leurent G. (2018). Percutaneous repair or medical treatment for secondary mitral regurgitation. N Engl J Med.

