# Supplementary material for: Forward Flow in Patients With Heart Failure and Functional Mitral Regurgitation: The COAPT Trial
Source: J Soc Cardiovasc Angiogr Interv. 2025 Mar 26;4(5):102609. doi: 10.1016/j.jscai.2025.102609 (PMC12126076; doi:10.1016/j.jscai.2025.102609)

**Supplemental Appendix**

**Forward Flow in Patients with Functional Mitral Regurgitation:**

**The COAPT Trial**

Zachary M. Gertz, MD, MBE; Philippe Pibarot, DVM, PhD; Zhipeng Zhou, MA; Michael J. Schonning, MS, MBS; Björn Redfors, MD, PhD; Yanru Li, MS, MPH; Saibal Kar, MD; D. Scott Lim, MD; Neil J. Weissman, MD; David J. Cohen, MD, MSc; JoAnn Lindenfeld, MD; William T. Abraham, MD; Michael J. Mack, MD; Federico M. Asch, MD; Gregg W. Stone, MD

| **Title** | **Page Number** |
| --- | --- |
| **Supplemental Table S1. Patient characteristics stratified by baseline forward flow tertile** | 2 |
| **Supplemental Table S2. Echocardiographic characteristics stratified by baseline forward flow tertile** | 3 |
| **Supplemental Table S3.** **Baseline patient characteristics stratified by whether forward flow increased from baseline to 30 days** | 4 |
| **Supplemental Table S4. Baseline echocardiographic characteristics stratified by whether forward flow increased from baseline to 30 days** | 5 |
| **Supplemental Table S5. Adverse events between 30 days and 2 years stratified by whether forward flow increased from baseline to 30 days and according to treatment group** | 6 |
| **Supplemental Figure S1. Consort diagram illustrating randomization according to forward flow tertiles** | 7 |
| **Supplemental Figure S2. Forward flow over time stratified by treatment group, without imputations for missing data** | 8 |

**Table S1. Patient characteristics stratified by baseline forward flow tertile**

|  | **First Tertile**  **N = 189** | **Second Tertile**  **N = 190** | **Third Tertile**  **N = 189** | **P value** |
| --- | --- | --- | --- | --- |
| Age (years) | 69.6 ± 11.4 | 72.9 ± 11.3 | 74.1 ± 10.5 | 0.0002 |
| Female sex (%) | 67 (35.4) | 62 (32.6) | 78 (41.3) | 0.20 |
| White race (%) | 126 (66.7) | 145 (76.3) | 151 (79.9) | 0.01 |
| Diabetes (%) | 72 (38.1) | 71 (37.4) | 68 (36.0) | 0.91 |
| Hypertension (%) | 148 (78.3) | 152 (80.0) | 156 (82.5) | 0.58 |
| Hypercholesterolemia (%) | 91 (48.1) | 102 (53.7) | 105 (55.6) | 0.32 |
| Previous myocardial infarction (%) | 80 (42.3) | 103 (54.2) | 109 (57.7) | 0.007 |
| Previous coronary artery bypass surgery (%) | 49 (25.9) | 77 (40.5) | 104 (55.0) | <0.0001 |
| Previous stroke or TIA (%) | 27 (14.3) | 38 (20.0) | 33 (17.5) | 0.34 |
| Peripheral vascular disease (%) | 25 (13.2) | 36 (18.9) | 39 (20.6) | 0.14 |
| COPD (%) | 44 (23.3) | 37 (19.5) | 49 (25.9) | 0.32 |
| Atrial fibrillation or flutter (%) | 101 (53.4) | 112 (58.9) | 96 (50.8) | 0.27 |
| Body mass index (kg/m^2^) | 26.5 (23.2, 29.8) | 25.7 (23.2, 29.2) | 26.0 (23.2, 29.9) | 0.58 |
| Creatinine Clearance* |  |  |  |  |
| Mean (mL/min) | n = 187  53.9 ± 26.8 | n = 189  46.9 ± 24.5 | n = 185  47.4 ± 28.8 | 0.02 |
| ≤60 (mL/min) (%) | 126/187 (67.4) | 147/189 (77.8) | 137/185 (74.1) | 0.07 |
| Anemia^⸸^ (%) | 31 (16.4) | 47 (24.7) | 53 (28.0) | 0.02 |
| STS Replacement Score |  |  |  |  |
| Heart failure etiology |  |  |  |  |
| Ischemic (%) | 98 (51.9) | 115 (60.5) | 132 (69.8) | 0.002 |
| Nonischemic (%) | 91 (48.1) | 75 (39.5) | 57 (30.2) | 0.002 |
| NYHA Class ≥3 (%) | 123 (65.1) | 115 (60.8) | 105 (55.6) | 0.17 |
| BNP (pg/mL) | n = 143  770.0 (379.1, 1323.0) | n = 126  600.5 (336.0, 1197.0) | n = 120  536.5 (321.0, 1155.0) | 0.12 |
| NT-proBNP (pg/mL) | n = 39  2816.00 (1770.00, 6980.00) | n = 51  4080.00 (2030.00, 6310.00) | n = 54  2850.50 (1489.00, 6094.00) | 0.47 |

Values represented as mean (SD), n (%), or median (1^st^ quartile, 3^rd^ quartile) where applicable. *Creatinine clearance calculated using the Cockcroft-Gault equation. ⸸Anemia defined as <13 in males and <12 in females. BNP denotes B-types natriuretic peptide; COPD, chronic obstructive pulmonary disease; NT-proBNP, N-terminal pro-B-type natriuretic peptide; NYHA, New York Heart Association; STS, Society of Thoracic Surgeons; TIA, transient ischemic attack

**Table S2. Echocardiographic characteristics stratified by baseline forward flow tertile**

|  | **First Tertile**  **N = 189** | **Second Tertile**  **N = 190** | **Third Tertile**  **N = 189** | **P value** |
| --- | --- | --- | --- | --- |
| MR Severity – (%) |  |  |  |  |
| Moderate to severe (3+) | 83 (43.9) | 99 (52.1) | 118 (62.4) | 0.001 |
| Severe (4+) | 106 (56.1) | 91 (47.9) | 71 (37. | 0.001 |
| Effective regurgitant orifice area* – (cm^2^) | n = 184  0.43 ± 0.16 | n = 185  0.41 ± 0.17 | n = 181  0.38 ± 0.12 | 0.002 |
| LVESD – (cm) | 5.6 ± 0.8 | n = 188  5.3 ± 0.9 | n = 188  5.0 ± 0.9 | <0.0001 |
| LVEDD – (cm) | 6.4 ± 0.7 | n = 189  6.2 ± 0.7 | n = 188  6.0 ± 0.7 | <0.0001 |
| LVESV – (mL) | n = 175  151.6 ± 59.8 | n = 179  135.6 ± 60.9 | n = 177  117.8 ± 50.1 | <0.0001 |
| LVEDV – (mL) | n = 175  207.1 ± 75.4 | n = 179  193.8 ± 73.3 | n = 177  177.6 ± 62.1 | 0.0005 |
| LVESV index – (mL/m^2^) | 2.9 ± 0.5 | n = 188  2.8 ± 0.5 | n = 188  2.7 ± 0.5 | <0.0001 |
| LVEDV index – (mL/m^2^) | 3.3 ± 0.4 | n = 189  3.3 ± 0.5 | n = 188  3.2 ± 0.5 | 0.12 |
| Left atrial volume – (mL) | n = 185  95.5 ± 45.2 | n = 184  88.9 ± 40.5 | n = 185  88.2 ± 34.4 | 0.16 |
| Left atrial volume index – (mL/m^2^) | n = 185  50.0 ± 24.7 | n = 184  47.0 ± 20.8 | n = 185  47.6 ± 18.4 | 0.37 |
| LVEF – (%) | n = 175  27.4 ± 7.3 | n = 180  31.4 ± 9.7 | n = 177  34.9 ± 9.6 | <0.0001 |
| LVEF >40% – (%) | 9 (5.1) | 41 (22.8) | 48 (27.1) | <0.0001 |
| LVEF ≤40% – (%) | 166 (94.9) | 139 (77.2) | 129 (72.9) | <0.0001 |
| RVSP – mmHg | n = 166  46.2 ± 14.4 | n = 164  44.3 ± 12.7 | n = 165  42.8 ± 13.6 | 0.08 |
| Regurgitant volume* – (mL/beat) | n =184  57.4 ± 21.4 | n = 185  60.7 ± 24.3 | n = 181  60.7 ± 19.1 | 0.24 |
| Regurgitant fraction – (%) | n = 127  41.7 ± 14.2 | n = 84  34.3 ± 12.2 | n = 42  26.0 ± 12.8 | <0.0001 |
| Tricuspid regurgitation ≥2+ (%) | n = 185  39 (21.1) | n = 187  27 (14.4) | n = 183  26 (14.2) | 0.13 |
| Forward stroke volume index – (mL/m^2^) | n = 189  17.9 ± 3.0 | n = 190  25.9 ± 2.3 | n = 189  36.6 ± 5.8 | <.0001 |

Values represented as mean ± SD or n (%) where applicable. *Calculated by PISA. LVEDD denotes left ventricular end diastolic diameter; LVESD, left ventricular end systolic diameter; LVEDV, left ventricular end diastolic volume; LVESV left ventricular end systolic volume; LVEF, left ventricular ejection fraction; MR, mitral regurgitation; PISA, proximal isovelocity surface area; RVSP, right ventricular systolic pressure.

**Table S3.** **Baseline patient characteristics stratified by whether forward flow increased from baseline to 30 days**

|  | **FSVi increased**  **(N=231)** | | | **FSVi unchanged or decreased**  **(N=230)** | | | | **Overall P value** |
| --- | --- | --- | --- | --- | --- | --- | --- | --- |
|  | **TEER + GDMT** | **GDMT alone** | **P value** | | **TEER + GDMT** | **GDMT alone** | **P value** |  |
| Age (years) | 71.2 ± 11.3 | 72.4 ± 10.5 | 0.43 | | 71.6 ± 12.4 | 72.1 ± 11.0 | 0.71 | 0.93 |
| Female sex (%) | 39 (32.0) | 33 (30.3) | 0.78 | | 43 (38.1) | 55 (47.0) | 0.17 | 0.01 |
| White race (%) | 86 (70.5) | 79 (72.5) | 0.74 | | 88 (77.9) | 86 (73.5) | 0.44 | 0.30 |
| Diabetes (%) | 43 (35.2) | 43 (39.4) | 0.51 | | 38 (33.6) | 40 (34.2) | 0.93 | 0.46 |
| Hypertension (%) | 95 (77.9) | 90 (82.6) | 0.37 | | 9 (80.5) | 93 (79.5) | 0.84 | 0.98 |
| Hypercholesterolemia (%) | 67 (54.9) | 52 (47.7) | 0.27 | | 56 (49.6) | 60 (51.3) | 0.79 | 0.82 |
| Previous myocardial infarction (%) | 69 (56.6) | 53 (48.6) | 0.23 | | 51 (45.1) | 62 (53.0) | 0.23 | 0.43 |
| Previous coronary artery bypass surgery (%) | 46 (37.7) | 41 (37.6) | 0.99 | | 44 (38.9) | 50 (42.7) | 0.56 | 0.48 |
| Previous stroke or TIA (%) | 21 (17.2) | 16 (14.7) | 0.60 | | 22 (19.5) | 22 (18.8) | 0.90 | 0.38 |
| Peripheral vascular disease (%) | 18 (14.8) | 21 (19.3) | 0.36 | | 26 (23.0) | 18 (15.4) | 0.14 | 0.53 |
| COPD (%) | 25 (20.5) | 23 (21.1) | 0.91 | | 22 (19.5) | 27 (23.1) | 0.50 | 0.89 |
| Atrial fibrillation or flutter (%) | 63 (51.6) | 63 (57.8) | 0.35 | | 70 (61.9) | 57 (48.7) | 0.04 | 0.88 |
| Body mass index (per 1 kg/m^2^) | 27.1 ± 5.1 | 27.6 ± 6.3 | 0.47 | | 27.0 ± 6.2 | 26.9 ± 6.3 | 0.91 | 0.51 |
| Creatinine clearance, mL/min* | 53.4 ± 28.4 | 47 ± 24.7 | 0.07 | | 50.3 ± 25.1 | 49.9 ± 28.1 | 0.90 | 0.90 |
| ≤60 mL/min (%) | 89/121 (73.6) | 79/108 (73.1) | 0.94 | | 78 (69.6) | 86 (74.1) | 0.45 | 0.73 |
| Anemia^⸸^ (%) | 23 (18.9) | 31 (28.4) | 0.09 | | 26 (23.0) | 26 (22.2) | 0.89 | 0.84 |
| STS replacement score | 6.6 ± 4.1 | 8.9 ± 6.0 | <0.0001 | | 7.7 (4.6) | 7.8 (6.1) | 0.94 | 0.87 |
| Heart failure etiology |  |  |  | |  |  |  |  |
| Ischemic (%) | 76 (62.3) | 61 (56.0) | 0.33 | | 67 (59.3) | 71 (60.7) | 0.83 | 0.88 |
| Non-ischemic (%) | 46 (37.7) | 48 (44.0) | 0.33 | | 46 (40.7) | 46 (39.3) | 0.83 | 0.88 |
| NYHA class ≥3 (%) | 65 (53.3) | 70 (64.8) | 0.08 | | 66 (58.4) | 71 (60.7) | 0.73 | 0.85 |
| BNP (pg/mL) | 591.0  (298.0, 1130.0) | 751.0  (380.1, 1178.5) | 0.26 | | 540.0  (323.0, 1520.0) | 624.0  (361.0, 1136.0) | 0.99 | 0.74 |
| NT-proBNP (pg/mL) | 3026.5  (2124.0, 5339.0) | 2093.0  (1334.0, 5919.5) | 0.34 | | 2351.0  (1640.0, 5745.0) | 2995.5  (1296.5, 5347.5) | 0.85 | 0.58 |

Values represented as n (%), mean ± SD, or median (1^st^ quartile, 3^rd^ quartile). *Creatinine clearance calculated using the Cockcroft-Gault equation. ⸸Anemia defined as <13 in males and <12 in females. BNP denotes B-types natriuretic peptide; COPD, chronic obstructive pulmonary disease; NT-proBNP, N-terminal pro-B-type natriuretic peptide; NYHA, New York Heart Association; STS, Society of Thoracic Surgeons; TIA, transient ischemic attack.

**Table S4. Baseline echocardiographic characteristics stratified by whether forward flow increased from baseline to 30 days**

|  | **FSVi increased**  **(N=231)** | | | **FSVi unchanged or decreased**  **(N=230)** | | | **Overall P value** |
| --- | --- | --- | --- | --- | --- | --- | --- |
|  | **TEER + GDMT** | **GDMT alone** | **P value** | **TEER + GDMT** | **GDMT alone** | **P value** |  |
| MR severity – (%) |  |  |  |  |  |  |  |
| Moderate to severe (3+) | 54 (44.3) | 52 (47.7) | 0.60 | 64 (56.6) | 75 (64.1) | 0.25 | 0.002 |
| Severe (4+) | 68 (55.7) | 57 (52.3) | 0.60 | 49 (43.4) | 42 (35.9) | 0.25 | 0.002 |
| Effective regurgitant orifice area* – (cm^2^) | 0.4 ± 0.2 | 0.4 ± 0.2 | 0.22 | 0.4 ± 0.1 | 0.4 ± 0.1 | 0.63 | 0.0003 |
| LVESD – (cm) | 5.4 ± 0.9 | 5.4 ± 0.8 | 0.80 | 5.2 ± 0.8 | 5.2 ± 1.0 | 0.56 | 0.01 |
| LVEDD – (cm) | 6.3 ± 0.7 | 6.2 ± 0.7 | 0.61 | 6.0 ± 0.7 | 6.2 ± 0.8 | 0.31 | 0.01 |
| LVESV – (mL) | 141.5 ± 59.0 | 139.9 ± 52.2 | 0.84 | 127.1 ± 53.1 | 132.6 ± 70.2 | 0.51 | 0.056 |
| LVEDV – (mL) | 200.7 ± 75.1 | 198.5 ± 62.8 | 0.82 | 185.3 ± 62.6 | 187.9 ± 84.7 | 0.79 | 0.059 |
| LVESV index – (mL/m^2^) | 73.9 ± 28.9 | 73.5 ± 27.7 | 0.91 | 67.5 ± 27.3 | 70.3 ± 33.2 | 0.50 | 0.09 |
| LVEDV index – (mL/m^2^) | 104.9 ± 35.7 | 103.1 ± 31.9 | 0.70 | 98.2 ± 31.2 | 99.9 ± 39.0 | 0.73 | 0.13 |
| Left atrial volume – (mL) | 91.4 ± 38.7 | 95.2 ± 49.2 | 0.52 | 87.8 ± 29.5 | 83.6 ± 30.2 | 0.29 | 0.03 |
| Left atrial volume index – (mL/m^2^) | 48.0 ± 19.8 | 49.9 ± 25.3 | 0.53 | 46.7 ± 16.0 | 44.9 ± 14.5 | 0.36 | 0.09 |
| LVEF – (%) | 30.3 ± 8.2 | 30.5 ± 9.4 | 0.89 | 32.7 ± 9.9 | 31.4 ± 10.0 | 0.37 | 0.07 |
| >40% – (%) | 15 (13.3) | 17 (17.0) | 0.45 | 25 (22.7) | 21 (18.9) | 0.49 | 0.12 |
| ≤40% – (%) | 98 (86.7) | 83 (83.0) | 0.45 | 85 (77.3) | 90 (81.1) | 0.49 | 0.12 |
| RVSP – mmHg | 44.5 ± 14.9 | 45.2 ± 13.4 | 0.73 | 44.2 ± 12.9 | 43.7 ± 14.9 | 0.81 | 0.50 |
| Regurgitant volume* – (mL/beat) | 60.1 ± 22.7 | 60.9 ± 22.7 | 0.79 | 58.3 ± 17.3 | 56.9 ± 17.1 | 0.55 | 0.13 |
| Regurgitant fraction – (%) | 39.2 ± 14.2 | 36.7 ± 12.7 | 0.19 | 35.9 ± 13.7 | 32.3 ± 17.3 | 0.31 | 0.04 |
| Tricuspid regurgitation ≥2+ (%) | 16 (13.2) | 11 (10.5) | 0.53 | 15 (13.4) | 19 (17.0) | 0.46 | 0.32 |
| Forward stroke volume index – (mL/m^2^) | 23.4 ± 6.6 | 23.4 ± 7.1 | 0.98 | 30.6 ± 9.4 | 31.0 ± 8.4 | 0.71 | <0.0001 |

Values represented as n (%) or mean ± SD. *Calculated by PISA. LVEDD denotes left ventricular end-diastolic diameter; LVESD, left ventricular end-systolic diameter; LVEDV, left ventricular end-diastolic volume; LVESV left ventricular end-systolic volume; LVEF, left ventricular ejection fraction; MR, mitral regurgitation; PISA, proximal isovelocity surface area; RVSP, right ventricular systolic pressure.

**Table S5. Adverse events between 30 days and 2 years stratified by whether forward flow increased from baseline to 30 days and according to treatment group**

|  | **Increased Forward Flow**  **N = 231** | | | **Decreased or Unchanged Forward Flow**  **N = 230** | | | **p-value for interaction** |
| --- | --- | --- | --- | --- | --- | --- | --- |
|  | TEER + GDMT | GDMT Alone | HR [95% CI] | TEER + GDMT | GDMT Alone | HR [95% CI] |  |
| All-cause death | 27 (22.3) | 32 (31.8) | 0.68 [0.41, 1.14] | 32 (28.9) | 47 (42.6) | 0.63 [0.40, 0.99] | 0.83 |
| CV cause | 25 (20.9) | 25 (25.9) | 0.81 [0.47, 1.41] | 24 (22.9) | 37 (35.0) | 0.60 [0.36, 1.01] | 0.45 |
| Related to HF | 12 (10.8) | 17 (18.5) | 0.57 [0.27, 1.19] | 9 (9.4) | 25 (25.0) | 0.34 [0.16, 0.72] | 0.33 |
| Not related to HF | 13 (11.3) | 8 (9.2) | 1.32 [0.55, 3.18] | 15 (14.9) | 12 (13.4) | 1.16 [0.54, 2.48] | 0.84 |
| Non-CV cause | 2 (1.7) | 7 (7.9) | 0.24 [0.05, 1.13] | 8 (7.8) | 10 (11.6) | 0.74 [0.29, 1.88] | 0.21 |
| All-cause hospitalization | 67 (61.0) | 75 (79.4) | 0.56 [0.41, 0.79] | 66 (66.4) | 74 (77.5) | 0.82 [0.59, 1.15] | 0.15 |
| CV cause | 50 (44.4) | 62 (63.8) | 0.57 [0.39, 0.83] | 49 (49.6) | 59 (59.8) | 0.73 [0.50, 1.06] | 0.43 |
| Related to HF | 35 (31.2) | 53 (54.7) | 0.46 [0.30, 0.71] | 33 (33.7) | 48 (47.8) | 0.56 [0.36, 0.87] | 0.60 |
| Not related to HF | 26 (23.9) | 23 (25.7) | 0.91 [0.52, 1.59] | 28 (29.0) | 32 (34.0) | 0.87 [0.53, 1.45] | 0.91 |
| Death or HFH | 46 (38.8) | 61 (60.5) | 0.53 [0.36, 0.77] | 49 (45.3) | 65 (61.9) | 0.60 [0.42, 0.88] | 0.64 |
| Death from CV cause or HF hospitalization | 45 (38.2) | 57 (57.9) | 0.55 [0.37, 0.82] | 43 (41.4) | 59 (56.9) | 0.59 [0.40, 0.87] | 0.87 |
| Major bleeding | 1 (0.9) | 0 (0.0) | N/A | 1 (1.2) | 0 (0.0) | N/A | 1.00 |
| Unplanned mitral-valve intervention | 1 (0.8) | 6 (8.1) | 0.13 [0.02, 1.70] | 4 (4.3) | 4 (4.8) | 0.93 [0.23, 3.72] | 0.14 |
| MitraClip^TM^ implantation | 1 (0.8) | 4 (6.1) | 0.19[0.02, 1.70] | 3 (3.3) | 2 (3.0) | 1.34 [0.22, 8.01] | 0.19 |
| Mitral-valve surgery | 0 (0.0) | 2 (2.1) | N/A | 1 (9.0) | 2 (1.7) | 0.51 [0.05, 5.64] | 1.00 |
| PCI or CABG | 0 (0.0) | 3 (3.6) | N/A | 4 (4.3) | 2 (2.1) | 1.91 [0.35, 10.44] | 1.00 |
| PCI | 0 (0.0) | 3 (3.6) | N/A | 4 (4.3) | 2 (2.1) | 1.91 [0.35, 10.44] | 1.00 |
| CABG | 0 (0.0) | 0 (0.0) | N/A | 0 (0.0) | 0 (0.0) | N/A | N/A |
| Neurological Event | 6 (5.5) | 7 (8.7) | 0.69 [0.23, 2.06] | 3 (3.0) | 10 (10.0) | 0.29 [0.08, 1.06] | 0.29 |
| Stroke | 5 (4.6) | 4 (5.4) | 1.00 [0.27, 3.72] | 2 (2.2) | 9 (9.2) | 0.21 [0.05, 0.98] | 0.11 |
| TIA | 1 (0.8) | 3 (3.4) | 0.28 [0.03, 2.66] | 2 (2.0) | 1 (0.9) | 2.01 [0.18, 22.22] | 0.24 |
| Myocardial infarction | 3 (2.7) | 7 (8.1) | 0.35 [0.09, 1.34] | 7 (7.2) | 6 (6.1) | 1.15 [0.39, 3.43] | 0.18 |
| New CRT | 4 (3.7) | 4 (4.6) | 0.79 [0.20, 3.16] | 1 (0.9) | 3 (2.6) | 0.34 [0.04, 3.24] | 0.49 |
| LVAD implantation or heart transplantation | 4 (4.0) | 3 (3.9) | 1.04 [0.23, 4.63] | 2 (2.4) | 9 (9.5) | 0.20 [0.04, 3.24] | 0.11 |
| LVAD implantation | 4 (4.0) | 3 (3.9) | 1.04 [0.23, 4.63] | 1 (1.2) | 5 (5.4) | 0.18 [0.02, 1.54] | 0.18 |
| Heart transplantation | 0 (0.0) | 0 (9.0) | N/A | 1 (1.2) | 5 (4.9) | 0.19 [0.02, 1.60] | 1.00 |
| RRT | 2 (1.9) | 10 (12.3) | 0.15 [0.03, 0.69] | 3 (3.4) | 6 (6.0( | 0.48 [0.12, 1.92] | 0.29 |

Data are presented as events (Kaplan-Meier %). CABG denotes coronary artery bypass grafting; CRT, cardiac resynchronization therapy; CV, cardiovascular; GDMT, guideline-directed medical therapy; HF, heart failure; HFH, heart failure hospitalization; LVAD, left ventricular assist device; PCI, percutaneous coronary intervention; RRT, renal replacement therapy; TEER, transcatheter-edge-to-edge repair; TIA, transient ischemic attack.

**Figure S1. Consort diagram illustrating randomization according to forward flow tertiles**

FSVI = forward stroke volume index; GDMT = guideline-directed medical therapy; TEER = transcatheter edge-to-edge repair

**
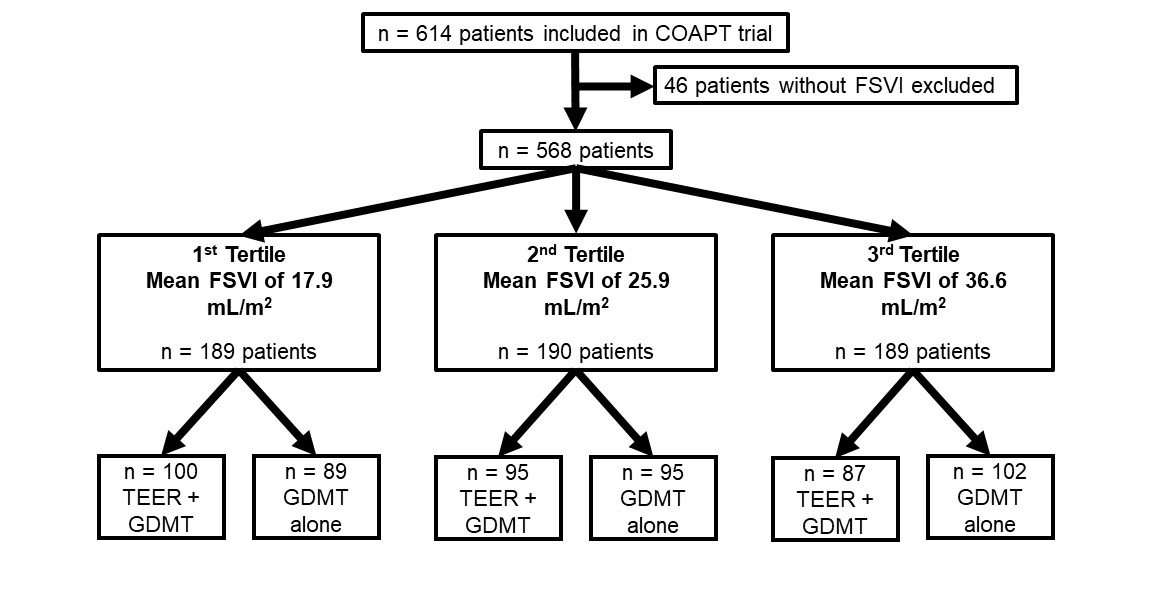
**

**Supplemental Figure S2. Forward flow over time stratified by treatment group, without imputations for missing data.**

Forward stroke volume index at baseline and at time points through 24 months, for the TEER plus GDMT and GDMT alone groups. At no time point did the between group difference, or the difference compared to baseline, reach statistical significance (p>0.05 for all comparisons).


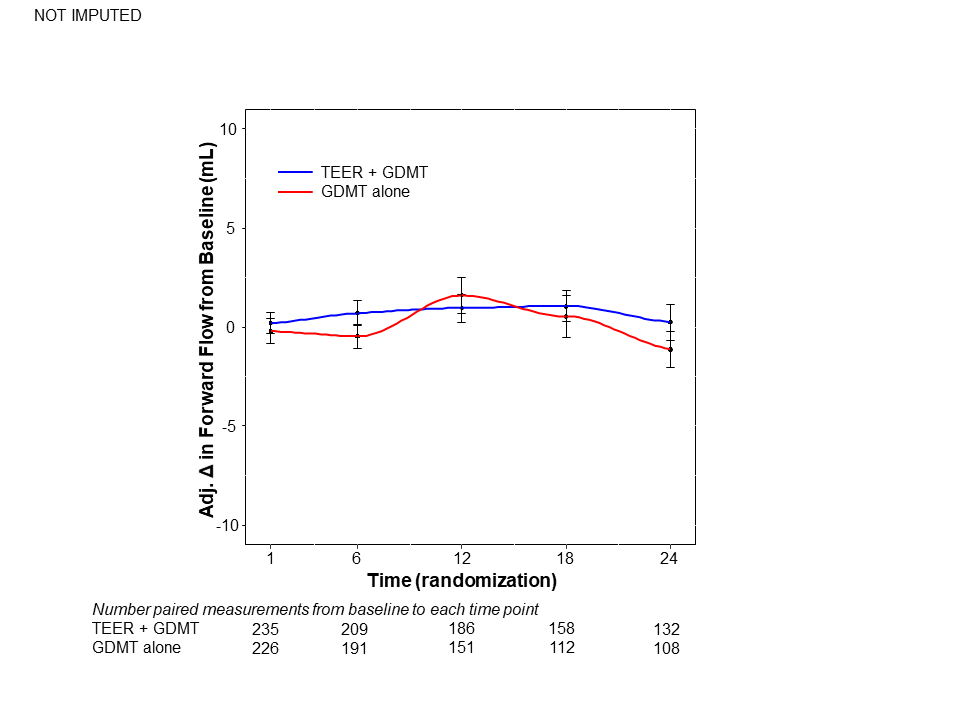

Supplement: Supplemental Tables and Figures [file mmc1.docx]
